# Development Perspectives for Curative Technologies in Primary Demyelinating Disorders of the Central Nervous System with Neuromyelitis Optica Spectrum Disorder (NMOSD) and Myelin Oligodendrocyte Glycoprotein Antibody-Associated Disease (MOGAD) at the Forefront

**DOI:** 10.3390/jpm14060599

**Published:** 2024-06-04

**Authors:** János György Pitter, László Nagy, Balázs Nagy, Rok Hren

**Affiliations:** 1 Syreon Research Institute, 1142 Budapest, Hungary; 2 Division of Pharmacoeconomics, Faculty of Pharmacy, University of Pecs, 7624 Pecs, Hungary; 3 Faculty of Mathematics and Physics, University of Ljubljana, 1000 Ljubljana, Slovenia; 4 Institute of Mathematics, Physics, and Mechanics, 1000 Ljubljana, Slovenia

**Keywords:** neuromyelitis optica spectrum disorder, myelin oligodendrocyte glycoprotein IgG-associated disease, multiple sclerosis, curative technology, immunotolerance, target patient selection, clinical development plan, cost-effectiveness, budget impact

## Abstract

Primary demyelinating disorders of the central nervous system (CNS) include multiple sclerosis and the orphan conditions neuromyelitis optica spectrum disorder (NMOSD) and myelin oligodendrocyte glycoprotein IgG-associated disease (MOGAD). Curative technologies under development aim to selectively block autoimmune reactions against specific autoantigens while preserving the responsiveness of the immune system to other antigens. Our analysis focused on target patient selection for such developments, carefully considering the relevant clinical, regulatory, and market-related aspects. We found that the selection of patients with orphan conditions as target populations offers several advantages. Treatments for orphan conditions are associated with limited production capacity, qualify for regulatory incentives, and may require significantly shorter and lower-scale clinical programs. Furthermore, they may meet a higher acceptable cost-effectiveness threshold in order to compensate for the low numbers of patients to be treated. Finally, curative technologies targeting orphan indications could enter less competitive markets with lower risk of generic price erosion and would benefit from additional market protection measures available only for orphan products. These advantages position orphan conditions and subgroups as the most attractive target indications among primary demyelinating disorders of the CNS. The authors believe that after successful proof-of-principle demonstrations in orphan conditions, broader autoimmune patient populations may also benefit from the success of these pioneering developments.

## 1. Introduction

The myelin sheath surrounding neural axons is essential for maintaining normal neuronal function. Damage to this myelin layer, known as demyelination, is a commonly observed pathological occurrence that results in various neurological symptoms. Primary demyelinating diseases initially affect the myelin sheath but often also lead to subsequent damage to the axons themselves [1]. In contrast, secondary demyelinating diseases arise from conditions that initially impact neurons or axons or from underlying factors that lead to consequential myelin damage and are typically observed in the elderly [2]. The most frequent primary demyelinating disorder is multiple sclerosis (MS), a leading cause of non-traumatic neurological disability among young individuals [3]. Other primary demyelinating disorders include neuromyelitis optica spectrum disorders (NMOSDs) and myelin oligodendrocyte glycoprotein IgG-associated disease (MOGAD), both of which are considered orphan diseases and typically manifest during early to middle adulthood [2,4,5].

Autoimmune reactions play a key role in the pathomechanism of primary demyelinating diseases. In MS, the autoimmune reaction is complex, involving both humoral and cellular processes and targeting several autoantigens, although some of these autoimmune reactions may be secondary to prior neural damage [6]. In NMOSD, anti-aquaporin 4 (the main water-channel protein in the CNS, which is mostly expressed on astrocytic end-feet) IgG is present in 70–90% of patients [7]. The immune response against this self-antigen could be triggered by a homologous peptide from Clostridium perfringens, a common species of human intestinal bacterial flora [6]. In MOGAD patients, antibodies binding to the native conformation of myelin oligodendrocyte glycoprotein are characteristic, although weak positivity has also been described in healthy populations, requiring a cut-off for diagnostic purposes. Besides serology findings, MRI results on spinal cord lesions are also helpful in distinguishing between the various forms of primary demyelinating disorders of the CNS [6,8].

Current treatment options for patients with MS, NMOSD, or MOGAD primarily include anti-inflammatory and immunosuppressive drugs, including biologicals (disease-modifying therapies) aimed at reducing inflammation and achieving immune suppression, respectively. However, these approaches also weaken immunity against pathogens and require life-long treatment, as they do not eliminate the autoimmune reactions by not restoring tolerance. More advanced medicinal technologies are under development with the goal of selectively blocking autoimmune reactions against specific autoantigens while preserving the responsiveness of the immune system to other antigens. As an early example, delivery of self-antigens with tolerogenic dendritic cell-based therapy to restore autoantigen tolerance reached the clinical development phase as early as 2015 in patients with rheumatoid arthritis [9], type 1 diabetes [10,11], and Crohn’s disease [12]. The use of tolerogenic dendritic cells in MS and NMOSD has also been investigated [13,14]. Other promising cell-based therapies are under development, aiming to restore tolerance to specific autoantigens via hematopoietic stem cell transplantation and the administration of regulatory T cells, mesenchymal stromal cells, or myeloid-derived suppressor cells [15,16]. The multitude of parallel research efforts to develop advanced medicinal technologies to restore specific immune tolerance are promising [17,18,19,20,21]. However, the high degrees of innovation and multi-disciplinarity inherent in these developments entail substantial development risks and costs. For many of these initiatives, external funding will eventually be required to ensure the financial sustainability of the research and development programs, including clinical studies, and to supplement any missing external knowledge whenever necessary. This is particularly pronounced for investigator-initiated academic developments. To enhance the attractiveness of additional funding opportunities, it is imperative to think from the perspective of potential investors and carefully consider economic aspects. Selecting the optimal target patient group for antigen-specific tolerance-restoring cell therapies is a critical step in this regard, both from a financial standpoint and in terms of early-phase technology development, where the specific autoantigen intended for immune tolerance restoration must be identified.

The aim of this analysis was to provide an overview of the options available for target patient selection in the context of curative cell-based therapy technologies developed for primary demyelinating disorders of the CNS, while carefully considering the relevant clinical, regulatory, and market-related factors. In this analysis, we focused on orphan diseases (NMOSD and MOGAD). However, we also emphasized the possibility of certain subsets of MS patients potentially being classified as orphan conditions. In this paper, we specifically examined clinical considerations, regulatory requirements pertaining to the clinical development programs, and market-related factors.

## 2. Clinical Considerations

### 2.1. Epidemiology

The incidences of MS, NMOSD and MOGAD are about 5, 0.4, and 0.2 in 100,000 person-years, respectively [7,22,23]. MS can be further stratified into subtypes based on the clinical course of the disease. The most common form is relapsing–remitting MS (RRMS), accounting for about 80–90% of incident cases; this subtype is characterized by acute episodes (relapses) separated by periods of remission without clinical worsening. Secondary progressive MS (SPMS), with a prevalence of 1–58 per 100,000 in the general population [24], is marked by a gradual worsening of symptoms independent of relapses. Conversely, primary progressive MS (PPMS), representing about 10–15% of all incident cases, manifests as a continuous aggravation of clinical symptoms without identifiable relapse episodes or remission periods [25,26]. Table 1 shows key epidemiological parameters for MS in comparison with AQP4 antibody-positive NMOSD (AQP4-IgG+ NMOSD) and MOGAD [27,28].

The incidence and prevalence of the selected target disease (sub)groups serve as the theoretical maximum for the number of patients who could potentially benefit from a newly developed technology. To encourage the development of new technologies for rare diseases, various public incentives have been introduced in both the EU and the US to compensate for the higher risk of insufficient financial returns for developers. Eligibility for orphan designation in the EU is contingent upon criteria such as a low prevalence in the population (<5/10,000), a clinically debilitating or life-threatening course, distinct medical or pharmacodynamic characteristics that limit off-label use, the absence of satisfactory alternative technologies in the EU, and clinically relevant results from preclinical or clinical tests conducted with the proposed new technology [29]. Similarly, in the US, the orphan designation criteria include a low prevalence in the US (affecting fewer than 200,000 persons) and a lack of profitability within 7 years after FDA approval [30]. Among primary demyelinating disorders of the central nervous system (CNS), several drugs developed to treat NMOSD have received orphan designation status in both the EU (eculizumab, satralizumab, and ravulizumab) [31,32] and the US (eculizumab, inebilizumab, ravulizumab, and satralizumab) [33]. Rituximab attained approval for NMOSD in Japan based on findings from an investigator-initiated phase II/III clinical study [34], yet it remains unapproved in other regions, including the EU and the US, where it is widely used as an off-label therapy for NMOSD patients [32]. In the US, orphan designations have been approved to treat either various symptoms (e.g., dalfampridine to improve walking and baclofen to treat intractable spasticity) or specific subgroups of MS patients (e.g., mitoxantrone to treat secondary progressive MS) [33]. In contrast, no orphan designation has been conferred thus far for the treatment of any subtype of MS and MOGAD patients in the EU [31]. Current obstacles in orphan drug development for rare variants of primary demyelinating disorders of the central nervous system (other than NMOSD) stem from the pharmaceutical industry’s risk aversion in clinical development and marketing due to small and heterogeneous patient populations [35,36]. On the other hand, existing orphan drug pricing and reimbursement policies have resulted in rapidly increasing public expenditure on orphan drugs in developed countries, accounting for about 5% and 10% of the total health budgets in the EU and the US, respectively [37]. It is unclear whether the overwhelming financial impact of successful orphan product development on national healthcare budgets hinders further positive orphan designation and marketing authorization decisions in the EU, as these actions are not made at the national level but at the European community level. In assessing the size of the target patient group, it is essential to consider factors beyond the overall market size and public incentives for orphan product development. Highly innovative technologies, such as personalized cell therapies, often entail substantial expenses for equipment, manufacturing procedures, and specialized personnel, resulting in high production costs per patient. This elevated treatment cost, coupled with a sizable target patient group, can lead to a significant healthcare budget impact that may prove socially unsustainable, even in high-income countries. Volume-based agreements with public healthcare payers may mitigate the budget impact of a new technology at the country level. However, this approach can inadvertently result in long waitlists for a new technology, raising concerns about equitable access to treatment. Insufficient or limited production capacity, which is common with cell therapies requiring personalized manufacturing, can also contribute to waitlists. Therefore, selecting a target patient group of an appropriate size—one that will not overwhelm the healthcare budget at the anticipated technology price and that can be feasibly served with the planned production capacity—is advisable. 

Furthermore, the homogeneity of the target patient group concerning the pathomechanism and clinical severity is another critical consideration. Targeting a broader patient population (assuming this is allowed by the budget and production capacity) can increase the market size and financial returns. However, targeting a heterogeneous group of patients can reduce clinical effectiveness and safety if the treatment is not properly matched to the pathomechanism and severity level. In primary demyelinating disorders of the CNS, selecting disorders for new therapies that aim to restore tolerance to autoantigens should consider the primary mechanism of damage to the myelin layer of axons (e.g., the role of cell-mediated or humoral adaptive immunity). In NMOSD, recently approved drugs (eculizumab, satralizumab, and inebilizumab) target a specific subgroup, i.e., NMOSD patients with aquaporin-4 IgG positivity (AQP4-IgG+ NMOSD). This ultra-orphan approach sets an attractive example for defining disease groups based on a shared pathomechanism, leading to more consistent clinical responses to new treatments. Another orphan example, MOGAD, is diagnosed based on the presence of a humoral immune response to myelin oligodendrocyte glycoprotein. Within MS, where both cell-mediated and humoral autoimmune processes contribute to myelin damage [6], there is an opportunity to identify orphan patient subgroups based on their autoimmune mechanisms; this approach remains unexplored in both the US and the EU. 

### 2.2. Unmet Clinical Need

Curative cell therapy technologies have the potential to restore immune tolerance to selected autoantigens in order to treat various autoimmune disorders. However, the high healthcare costs associated with personalized cell-based therapy can only be justified for conditions with limited treatment options and high unmet medical need. Hence, autoimmune disorders that can be managed through hormone replacement (e.g., type 1 diabetes, Addison’s disease, and Hashimoto’s thyroiditis) or dietary interventions (e.g., gluten-sensitive enteropathy and celiac disease) may not be suitable targets for curative cell-based therapy technologies compared to conditions with seriously debilitating or life-threatening courses despite current treatments. Orphan conditions in the EU fall into the latter category since high unmet need is a prerequisite for orphan designation [29]. Progressive forms of MS also have a severe clinical course with substantial unmet need. 

In most cases, MS initially manifests as clinically isolated syndrome (CIS), which presents as a single acute episode that is challenging to distinguish from other acute or subacute neurological diseases [38,39]. Various recognized risk factors contribute to the conversion of CIS to clinically definite MS, including age, gender, schooling, motor symptoms, imaging findings (infratentorial and periventricular lesions), and oligoclonal bands in the cerebrospinal fluid [38,40]. Additionally, radiologically isolated syndrome (RIS) patients may show no apparent neurological symptoms but display MRI signs of ongoing demyelination in the CNS [41]. Identified risk factors for the occurrence of a first clinical event in RIS patients include specific lesion patterns (the presence of infratentorial, spinal cord, or gadolinium-enhancing lesions) and cerebrospinal fluid-specific oligoclonal bands [42]. The presence of cerebrospinal fluid-specific oligoclonal bands is associated with a higher risk of progression from CIS and RIS to MS, underscoring the importance of humoral autoimmune processes in the initial stages of MS. When evaluating the predictive value of four risk factors together (age below 38 years, presence of oligoclonal bands in the cerebrospinal fluid or an increased IgG index, infratentorial lesions, and spinal cord lesions), the 10-year risk for the first clinical event was estimated to be 29% in cases with zero or one risk factor and 54%, 68%, and 87% in RIS subjects with two, three, or all four risk factors, respectively [43]. The median times from RIS diagnosis to the first clinical event were remarkably long at about 15 years, 8 years, 5 years, and 2.5 years in these groups [43]. However, most of the acute episodes occurred in patients with only two or three risk factors. 

Despite recent advances in understanding the pathogenesis of NMOSD, significant clinical needs remain unmet. While novel targeted therapies have emerged for patients with AQP4-IgG+ NMOSD, challenges persist in comprehending the long-term disease course, determining optimal durations of immunotherapy, devising strategies for treatment cessation and de-escalation, and identifying biomarkers indicative of attack risk [32]. Recently, the Neuromyelitis Optica Study Group (NEMOS) [44] recommended that the diagnosis of NMOSD should follow the revised criteria proposed by the International Panel for NMO Diagnosis (IPND) in 2015. They emphasized the importance of using up-to-date, standardized cell-based assays (CBAs), as both fixed and live CBAs are highly specific and sensitive for detecting AQP4-IgG [44]. Special care is needed to rule out differential diagnoses, especially in seronegative NMOSD patients, with key differential diagnoses including MOGAD, MS, neurosarcoidosis, paraneoplastic neurological syndromes, and infectious diseases [44]. In a follow-up publication [32], the NEMOS identified the treatment and prevention of attacks as essential pillars of NMOSD therapy to minimize the accrual of neurological disability. NMOSD differs from MS because neurological disability mainly results from poor and incomplete recovery from clinical attacks. They stated that long-term immunotherapy must be offered to patients with AQP4-IgG+ NMOSD after the first attack and concluded that four preventive immunotherapies approved for AQP4-IgG+ NMOSD (eculizumab, ravulizumab, inebilizumab, and satralizumab) “provide a more personalized approach to treatment, considering factors, such as disease activity, age, comorbidities, family planning, side effects, route of administration, patient choice, availability, and costs” [32]. 

In their recommendation [32], the NEMOS discussed long-term treatment options for AQP4-IgG+ NMOSD, including the monoclonal antibodies outlined in Table 2. Eculizumab works by inhibiting the complement cascade, which is involved in the inflammatory process of NMOSD. Administered intravenously, eculizumab requires regular infusions once every 2 weeks after the initiation of the treatment. Similar to eculizumab, ravulizumab is a long-acting complement factor C5 inhibitor that is also administered intravenously but requires less frequent dosing (once every 8 weeks after the initiation of the treatment) compared to eculizumab, enhancing patient convenience and adherence. Inebilizumab, a monoclonal antibody targeting CD19 on B cells, reduces the number of B cells that can produce AQP4-IgG. Administered via intravenous infusions once every 6 months after the initiation of the treatment, it offers a less frequent dosing schedule. Satralizumab, an interleukin-6 (IL-6) receptor inhibitor, can be self-administered subcutaneously once every 4 weeks after the initiation of the treatment. The NEMOS [32] found no high-level evidence demonstrating the superiority of one monoclonal antibody over another. They recommended initiating long-term immunotherapy in AQP4-IgG+ NMOSD with one of the monoclonal antibodies as a monotherapy, unless comorbidity necessitates combination therapy with classical immunosuppressive therapies [32]. The choice of immunotherapy should be based on factors such as attack severity, recovery from attacks, efficacy, onset of action, comorbidities, side effects, drug-related mortality, age, family planning, patient preferences, adherence, clinical utility, availability, and costs [32].

Although not specifically approved for NMOSD outside of Japan, rituximab is widely used off-label and targets CD20 on B cells, leading to B-cell depletion. Tocilizumab, an IL-6 receptor inhibitor, is also used off-label for NMOSD and has been increasingly utilized as a rescue therapy [32]. Rituximab is administered intravenously every 6 months after the initiation of the treatment, while tocilizumab is administered every 4–6 weeks, with the option to switch to subcutaneous application.

Double-negative patients with NMOSD test negative for both aquaporin-4 antibodies (AQP4-IgG) and myelin oligodendrocyte glycoprotein antibodies (MOG-IgG) [45]. In these cases, other CNS inflammatory diseases such as MS and neurosarcoidosis must be excluded. Currently, it is unclear whether double-negative NMOSD represents a single disease entity or a collection of pathogenetically diverse conditions, necessitating further investigations [32].

MOGAD shares clinical and radiological characteristics with other demyelinating diseases like MS and NMOSD. Diagnosis currently relies on the detection of antibodies against MOG (MOG-IgG) in serum or cerebrospinal fluid. Despite the introduction of MOG antibody (MOG-Ig) diagnostic testing, MOGAD diagnosis remains challenging due to its heterogeneous disease course, clinical presentations that overlap with other CNS demyelinating diseases, and limited awareness of MOGAD among healthcare providers [46]. 

The concept of disease-stopping therapies holds the potential to initiate treatment in the early clinical or even preclinical stages of a disease, aiming for complete healing and prevention of the progression of aggravating residual symptoms through multiple relapses and remission phases. While this ambition is medically compelling, it must also contend with economic health considerations. Developing new technologies for pre-symptomatic subjects or patients with mild, early-stage clinical symptoms necessitates accurately identifying high-risk subgroups expected to experience rapid disease progression. It is crucial to demonstrate an acceptable benefit–risk ratio based on the cost-effectiveness of a technology and its impact on healthcare budgets [42]. Identifying high-risk subgroups with high specificity and similar pathomechanisms may also enable defining new orphan conditions and guide the development of disease-stopping technologies, including tailored curative cell therapies. 

## 3. Regulatory Requirements of the Clinical Development Program

While a comprehensive overview of all relevant regulatory requirements in the EU and the US regarding the quality, nonclinical and clinical efficacy, and safety of emerging curative cell technologies would be extensive, our analysis focuses on the regulatory requirements of clinical development programs. This phase dominates the resource requirements of new health technology development projects, both in terms of time and financial resources.

Regulatory guidelines covering the clinical development of medicinal products offer valuable insights into regulatory requirements. Both the US and the EU provide general guidance on the planning, execution, and reporting of clinical trials, with harmonized guidelines available within the framework of the International Council for Harmonisation (ICH) at www.ich.org (accessed on 31 May 2024). Disease-specific guidelines from the EMA typically prioritize diseases affecting larger populations or attracting multiple development projects, leaving most orphan products without specific support. Instead, both the FDA and EMA provide tailored scientific advice and even assistance with protocol development for parties working on orphan developments. As expected, no specific guidelines were found for the clinical development of new medical technologies targeting patients with NMOSD or MOGAD. In contrast, the EMA has released a disease-specific guideline for the clinical investigation of medicinal products for the treatment of MS [47]. This guideline offers specific recommendations for the development of treatments modifying the natural course of the disease, which will also be relevant for future curative treatments with some modifications. For instance, considerations regarding the risk of opportunistic infections or malignancies due to general immunosuppressive states induced by disease-modifying therapies are less pertinent in the benefit–risk assessments of new treatments employing selective immunotolerance induction. 

Interestingly, the EMA guideline on MS explicitly advises against developing products for early-stage patients with CIS or RIS who do not meet the criteria for an MS diagnosis and highlights the importance of scientific discussion to confirm the usefulness of developed products [47]. The authors suggest that this cautionary stance reflects the typically slow clinical progression of CIS and RIS patients. Accurately identifying high-risk subgroups within CIS and RIS patients, characterized by early and rapid progression to severe MS, could significantly alter this perspective, potentially opening avenues for such developments.

In addition to regulatory guidelines, public assessment reports of new authorized treatments provide additional insight into disease-specific regulatory requirements. These reports, released by regulatory authorities as part of the authorization process, provide comprehensive summaries of the submitted evidence packages, including detailed overviews of the clinical development programs. European Public Assessment Reports (EPARs) of products authorized by the EMA can be searched by product name at www.ema.europa.eu (accessed on 31 May 2024), and public medical reviews on products authorized in the USA can be browsed at https://www.accessdata.fda.gov/scripts/cder/daf/index.cfm (accessed on 31 May 2024). Based on these reports, Table 2 summarizes the key characteristics of clinical development programs for recently approved treatments targeting primary demyelinating disorders of the CNS. 

The data presented in Table 2 indicate a common trend in the clinical development of new therapies for MS, where extensive phase 2 and phase 3 studies involving thousands of patients are standard. In contrast, clinical development programs for an orphan condition with a similar pathomechanism and disease severity (NMOSD) have often bypassed phase 2 studies. Instead, these programs have investigated dose–response characteristics as early as phase 1 in healthy volunteers, utilizing pharmacodynamic endpoints. This streamlined approach has expedited clinical development in NMOSD, considering the shorter recruitment periods and follow-up durations compared to typical phase II MS studies. Phase 3 programs for drugs targeting NMOSD have enrolled about one order of magnitude fewer patients than those for MS, yet they have identified a positive benefit–risk ratio based on clinical studies involving around 200 patients in total. In contrast, demonstrating an acceptable benefit–risk profile for MS drugs required larger studies involving around 2000 patients. Importantly, the durations of the follow-up periods were comparable between these indications, likely reflecting similar expectations of the study sponsors regarding relapse rates, which were the primary outcomes in nearly all phase III studies for both MS and NMOSD.

Clarification of the regulatory requirements for the clinical development of new technologies targeting MOGAD awaits the first marketing authorizations for this indication. However, considering MOGAD’s likely eligibility for orphan designation based on its epidemiology, clinical severity, and unmet medical need (no approved treatments at present), it is presumed that the clinical regulatory requirements for MOGAD will be similar to those for NMOSD. 

## 4. Market-Related Considerations

When assessing the financial implications of a newly developed technology, intellectual property considerations hold significant weight, as they directly impact the potential return on investment. The competitive landscape within the market heavily influences this potential, and in the case of RRMS, the market is already saturated with several competitors. As market exclusivity and patent protections will expire for more authorized products within the MS market, ongoing and swift price erosion will likely occur due to the presence of multiple generic and me-too alternatives. This scenario diminishes the overall financial potential of a developed technology. It is noteworthy that although various therapeutic agents are currently available for relapse prevention in RRMS, there are exceptionally few drugs aimed at delaying disability progression in progressive MS (PMS). Presently, ocrelizumab, a monoclonal antibody, is the only approved treatment for PPMS [48], while siponimod, a small-molecule drug, is approved for SPMS [49]. As mentioned by Sapko et al. [50], treating PMS is particularly challenging due to its complex pathogenesis, which shifts from primarily inflammatory to predominantly neurodegenerative processes with age and disease duration [50,51]. Consequently, the window for effective use of anti-inflammatory treatment is very narrow, emphasizing the need for neuroprotective and remyelinating therapies [50], although trials have shown inconclusive results so far [50,51].

However, compared to RRMS, the situation differs for AQP4-IgG+ NMOSD, as the first EU-approved product for this indication entered the market in 2019. Orphan products benefit from ten years of market protection in the EU, allowing them to remain the sole product on the market without competition from generic alternative competitors or similar medicines with comparable indications. With these additional market protection measures, it is unlikely that generic price erosion will impact NMOSD until at least 2029, even for the first approved product with this indication. This provides a more stable commercial landscape and potential financial returns for new technologies targeting this orphan condition. In the EU, no approved drugs are available for the treatment of MOGAD, creating an attractive competitive landscape for this indication.

For curative technologies targeting primary demyelinating disorders of the CNS, the justifiable price level is determined by the additional clinical and health system benefits compared to current gold-standard therapies [52]. Even in a crowded market like MS, a first-in-class curative technology capable of selectively restoring immunotolerance for a key autoantigen while circumventing the typical side effects associated with general immunosuppression from current disease-modifying therapies (e.g., opportunistic infections) may demonstrate favorable cost-effectiveness and a manageable budget impact. These factors are crucial prerequisites for securing public reimbursement and health system uptake in most European countries. For orphan indications, where patient numbers are limited, the willingness-to-pay threshold for achieving acceptable cost-effectiveness is higher, potentially resulting in more space to maneuver related to public reimbursement and health system uptake. Nevertheless, accurate predictive identification of narrow patient subgroups within CIS, RIS, or MS with early and rapid progression to severe MS could facilitate the delineation of new orphan conditions within the MS spectrum. These conditions would then become eligible for regulatory and public reimbursement benefits similar to those seen for NMOSD and MOGAD. 

In the US, where a diverse array of health insurance providers and packages are available, citizens are offered a range of options. However, it is important to note that the decision-making processes may not always be fully informed, resulting in various levels of understanding among individuals about available offerings. The early inclusion of new treatments often enhances the appeal of more prestigious and higher-cost insurance plans, as individuals seek access to cutting-edge healthcare options for themselves and their families. Accordingly, the tendency for rapid inclusion of any new technology in competitive but expensive health insurance plans could facilitate market access to curative cell therapies in the US across a wide range of medical indications. 

## 5. Conclusions

Our analysis focused on the options for target patient groups in the development of curative cell technologies for primary demyelinating disorders of the CNS, carefully considering the relevant clinical, regulatory, and market-related aspects, in order to support indication selection in the EU-funded IMMUTOL project at https://immutol-horizon.eu/ (accessed on 31 May 2024). In this study, we acknowledged the perspective of potential investors and to that end considered economic aspects. Given the personalized manufacturing required for cell therapies, significant manufacturing costs and limited production capacity are anticipated. These factors create notable barriers to treating large patient populations without the risk of lengthy waitlists and concerns regarding equitable access to care. Besides MS, primary demyelinating disorders of the CNS include the designated orphan indication AQP4-IgG+ NMOSD and the possible orphan designation candidate MOGAD, which have similar epidemiology, clinical severity, and unmet needs. Beyond NMOSD and MOGAD, narrow patient subgroups within MS, such as those with CIS or RIS who display early and rapid progression to severe MS, could also be eligible for orphan designation. 

The selection of patients with orphan conditions as target populations for curative technologies for primary demyelinating disorders of the CNS offers several advantages. Treatments for orphan conditions are associated with limited production capacity, qualify for regulatory incentives including protocol assistance, can be developed in significantly shorter and smaller-scale clinical programs, and may meet a higher acceptable cost-effectiveness threshold. Furthermore, curative cell technologies targeting orphan indications could enter less competitive markets with lower risk of generic price erosion and would benefit from additional market protection measures available only for orphan products, including ten years of market protection in the EU against all similar products with similar indications (Table 3). These advantages position orphan conditions and subgroups as the most attractive target indications among primary demyelinating disorders of the CNS, surpassing MS as a whole disease entity: a proverbial high-hanging and over-ripened fruit. 

## 6. Future Directions

The ongoing advancements in curative cell therapies, aimed at reinstating immunotolerance to specific autoantigens while preserving immune system responsiveness to non-self antigens, give hope to all patients suffering from autoimmune diseases, along with their caregivers and healthcare providers. Initiation of development projects is essential to validate the proofs of concept of these innovative approaches for select autoimmune indications characterized by homogeneous pathomechanisms and clinical profiles. Orphan indications, as discussed in our analysis, are particularly promising for these pioneering exercises, capitalizing on available regulatory, patient access, and market protection incentives for orphan developments. However, following successful developments in carefully selected orphan indications, subsequent steps must encompass additional efforts to make these transformative technologies accessible to broader patient populations at socially sustainable prices. In this post-proof-of-principle research phase, scaling the technology becomes imperative to minimize the manufacturing costs and increase the production capacity of personalized cell therapies while upholding stringent product quality standards. A pertinent analogy lies in the significant reduction in the cost of sequencing a million base pairs of DNA, which plummeted from USD 1000 to USD 0.1 within a decade [53]. We can only hope that after successful proof-of-principle demonstrations in selected narrow patient groups, broader autoimmune patient populations whose conditions are controlled by current treatment options but still face significant residual impairment of their quality of life can also benefit from these technological breakthroughs. Possible examples include type 1 diabetes patients who require lifelong daily diet control and insulin replacement and celiac disease patients with strict dietary restrictions. The ultimate vision transcending the current developments is nothing short of rendering personalized cell therapies accessible for routine clinical practice across all autoimmune diseases characterized by suitable pathomechanisms.

## Figures and Tables

**Table 1:** Key epidemiological parameters for multiple sclerosis (MS), AQP4 antibody-positive NMOSD (AQP4-IgG+ NMOSD), and myelin oligodendrocyte glycoprotein IgG-associated disease (MOGAD) [27,28].

	MS	AQP4-IgG+ NMOSD	MOGAD
Female-to-male ratio	2–4 to 1	9 to 1	1 to 1
Prevalence	Up to 100–200/100,000 in white populations, less than 5–50/100,000 in many non-white populations	1/100,000 in white populations, 3.5/100,000 in East Asian populations, and from 1.8 to 10/100,000 in black populations	1.3–2.5/100,000
Incidence	Up to 100/million in white populations, with incidence decreasing with decreasing latitude	Around 0.5–0.8/million in white populations, higher in non-white populations	3.4–4.8/million

**Table 2:** Key characteristics of full clinical development programs of new treatments for primary demyelinating disorders of the central nervous system.

	MS	AQP4-IgG+ NMOSD	MOGAD
Recently approved drugs (EMA) ^1^	ublituximab (2023) ponesimod (2021) ofatumumab (2021) ozanimod (2020) ocrelizumab (2018) cladribine (2017)	ravulizumab (2023) inebilizumab (2022) satralizumab (2021) eculizumab (2019)	none
Phase II studies	~200 patients FU: 0.5–2 years	none (dose finding in phase I)	-
Phase III studies	877 to 2666 patients FU: 2–4 years	143 to 230 patients FU: 3–4 years	-

^1^ Authorized in the last 7 years. FU, follow-up; patient numbers indicate the total size of the study population(s) in the corresponding clinical studies; MS, multiple sclerosis; AQP4-IgG+ NMOSD, AQP4 antibody-positive NMOSD; MOGAD, myelin oligodendrocyte glycoprotein IgG-associated disease.

**Table 3:** Overview of possible target groups for new treatments for primary demyelinating disorders of the central nervous system.

	MS as a Whole	NMOSD	MOGAD	High-Risk CIS or RIS Patients with Rapid Progression to Severe MS
Incidence in 100,000 person-years	~5	~0.4	~0.2	subject to subgroup definition
Orphan status	Not eligible	Designated	Eligible	Can be eligible
CHMP guideline on clinical development	Yes [47]	No	No	Yes [47] (not endorsed without scientific advice)
Precedent EU developments	>10 drugs	3 drugs (AQP4-IgG+)	0	0
Phase II	>200 patients	skipped	no data; requirements are presumably similar to NMOSD	
Phase III	>1000 patients	~200 patients		
Competitor landscape	Huge	Some	None	None
Expected price erosion	Extremely high	Medium/Low	Low	Low

## Data Availability

The data presented in this study are available upon request from the corresponding author.

## Abbreviations

AQP4-IgG+ NMOSD, aquaporin-4 IgG-positive neuromyelitis optica spectrum disorder; CIS, clinically isolated syndrome; CNS, central nervous system; EMA, European Medicines Agency; FDA, Food and Drug Agency; MOGAD, myelin oligodendrocyte glycoprotein IgG-associated disease; NMOSD, neuromyelitis optica spectrum disorder; MS, multiple sclerosis; RIS, radiologically isolated syndrome.
